# Morte Súbita em Lactante Portadora de Prolapso da Válvula Mitral Arritmogênico

**DOI:** 10.36660/abc.20230358

**Published:** 2024-03-14

**Authors:** Erika Olivier Vilela Bragança, Fabio Luis Valério da Silva

**Affiliations:** 1 RitmoCheck São José dos Campos SP Brasil RitmoCheck, São José dos Campos, SP – Brasil

**Keywords:** Prolapso da Válvula Mitral, Morte Súbita, Arritmia

## Introdução

O prolapso da válvula mitral (PVM) é a doença cardíaca valvar mais comum, afetando de 2 a 3% da população geral e é definido pelo deslocamento sistólico de um ou ambos os folhetos da válvula mitral ≥2 mm acima do plano do anel mitral na visão sagital da válvula mitral ao ecocardiograma.^1^ Os desfechos do PVM sem regurgitação, em geral, são benignos; no entanto, um pequeno subgrupo mal definido de indivíduos apresenta alto risco de arritmias malignas e morte súbita (MS). Dados de autópsia mostram uma prevalência de 4 a 7% de PVM em jovens com MS.^2^ Devido à baixa taxa de evento e à falta de coortes com um número robusto de pacientes, acessar a incidência precisa de MS e PVM na população geral e em subgrupos de pacientes permanece um desafio. O diagnóstico de PVM arritmogênico (PVMA) é feito na presença de PVM e arritmia ventricular, considerando densidade de arritmia ventricular ≥5% em Holter de eletrocardiograma (ECG) de 24h ou a presença de arritmia complexa – taquicardia ventricular não sustentada (TVNS), taquicardia ventricular (TV) ou fibrilação ventricular (FV) e na ausência de outro substrato arritmogênico.^3^

### Descrição

**Figura 1:**
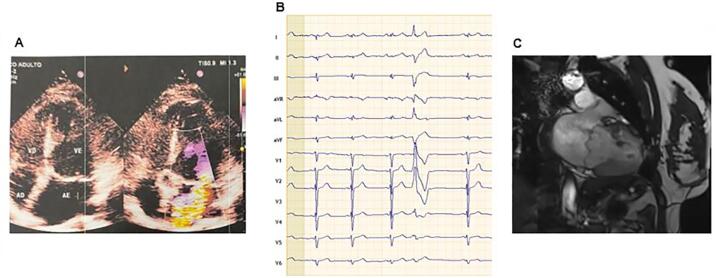
– A) Ecocardiograma: doppler colorido da válvula mitral com importante regurgitação. Observa-se dilatação ventricular; B) Eletrocardiograma de 12 derivações: ritmo sinusal, bloqueio interatrial de 1 grau (duração de onda P 125 ms), fragmentação de onda R em derivações D1 e aVL, intervalo QTc de 420 ms, ectopia ventricular com morfologia de bloqueio de ramo direito, com eixo superior (D2 e D3 negativos), complexo QRS da ectopia predominantemente negativo em derivação V5 e duração do QRS da ectopia de 195 ms - sugestivo de origem em músculo papilar posterior; C) Ressonância magnética de coração: importante dilatação esquerda, prolapso da válvula mitral com regurgitação severa e disjunção do anel mitral.

Relatamos o caso de JAS, paciente do sexo feminino, 27 anos, casada e internada 13 meses atrás após parada cardiorrespiratória (PCR) com relatório de alta hospitalar que informa “PCR em ritmo chocável e reanimada com sucesso”. O relatório informa ainda internação por 18 dias, realização de ECGs sem alterações significativas, ecocardiograma com dilatação leve a moderada de átrio esquerdo, válvula mitral mixomatosa com prolapso significativo de suas cúspides associada a insuficiência valvar moderada, dilatação ventricular esquerda moderada com função de ventrículo esquerdo preservada (diâmetro do átrio esquerdo de 45 mm, diâmetro diastólico do ventrículo esquerdo de 66 mm e fração de ejeção do ventrículo esquerdo de 67%) ( Figura 1A ) e cinecoronariografia sem lesões obstrutivas. O relatório conclui que “não há necessidade de prolongar internação para investigação adicional e PCR de causa indeterminada após investigação”. A paciente recebeu alta hospitalar sem medicações e foi orientada a procurar o posto de saúde para acompanhamento. Apresentou, como intercorrências durante a internação, pneumonia broncoaspirativa, insuficiência renal que exigiu hemodiálise, e acidente vascular cerebral isquêmico em região parietal esquerda.

Após a alta, apresentou ciclos menstruais irregulares, não fazia controle de natalidade e engravidou. Era primípara, G_1_P_0_A_0_, e a gestação transcorreu sem anormalidades. Foi submetida a parto cesariana com 38 semanas por indicação do cardiologista, e deu à luz a um bebê do sexo feminino, pesando 2.600 gramas, com altura de 47 centímetros, e Apgar 8 e 10. A alta hospitalar ocorreu em 48 horas. Veio na primeira e única consulta com a lactente de 2 semanas, em aleitamento materno, acompanhada da sobrinha de 13 anos e com queixa de cansaço aos moderados esforços. Como antecedentes pessoais, relatou arritmia desde a infância e os demais supracitados em relatório de alta hospitalar. Não apresentava antecedentes familiares relevantes, não era tabagista e/ou etilista, não praticava exercícios, negava outras cirurgias, não teve COVID-19 e não fazia uso regular de medicação. No exame físico chamava a atenção que a paciente estava em aleitamento materno e à ausculta cardíaca detectou-se um sopro sistólico em foco mitral +/4+ com irradiação para axila e o restante era normal. O ECG mostrava um ritmo sinusal, bloqueio interatrial de 1 grau (duração de onda P 125 ms), fragmentação de onda R em derivações D1 e aVL, intervalo QTc de 420 ms, ectopia ventricular com morfologia de bloqueio de ramo direito, com eixo superior (D2 e D3 negativos), complexo QRS da ectopia predominantemente negativo em derivação V5 e duração do QRS da ectopia de 195 ms, o que é sugestivo de origem em músculo papilar posterior ( Figura 1B ). Trazia o ecocardiograma transtorácico já descrito anteriormente e a imagem era sugestiva de regurgitação mitral importante com dilatação de ventrículo esquerdo ( Figura 1A ). Também portava um Holter de ECG de 24h com frequências que variaram mínima 42 bpm, média 68 bpm e máxima 113 bpm, 2.449 (3%) ectopias ventriculares bimórficas, das quais 2.428 eram isoladas, com 9 pares e 1 TVNS de 3 batimentos e frequência de 121 bpm, 1 ectopia atrial era isolada, com alterações difusas da repolarização ventricular com prolongamento do intervalo QTc (QTc máximo 500 ms) e sem correlação entre os sintomas e alterações eletrocardiográficas. A hipótese diagnostica foi de PVMA em paciente recuperada de MS e o cardiodesfibrilador (CDI) e reparo da válvula mitral foram indicados. A paciente recusou o tratamento, a despeito de todas as orientações dadas, e solicitou retornar com o marido em consulta agendada. Foi iniciado o tratamento com betabloqueador e foi solicitada ressonância magnética (RM) de coração. Na data da consulta, 1 mês depois, a paciente remarcou, solicitando mais um mês para retornar. Uma semana antes da data agendada a paciente apresentou novo quadro de PCR e tentou-se realizar a reanimação sem sucesso. Antes da consulta, a paciente havia realizado a RM de coração ( Figura 1C ). O laudo foi obtido *post mortem* na clínica que realizou o exame e mostrava aumento do átrio esquerdo, dilatação do ventrículo esquerdo associado a PVM e disjunção do anel mitral (DAM), regurgitação mitral grave e excêntrica, e aumento do trabeculado subendocárdico associado a fibrose subendocárdica ântero e inferolateral basal e medial.

## Discussão

**Figura 2:**
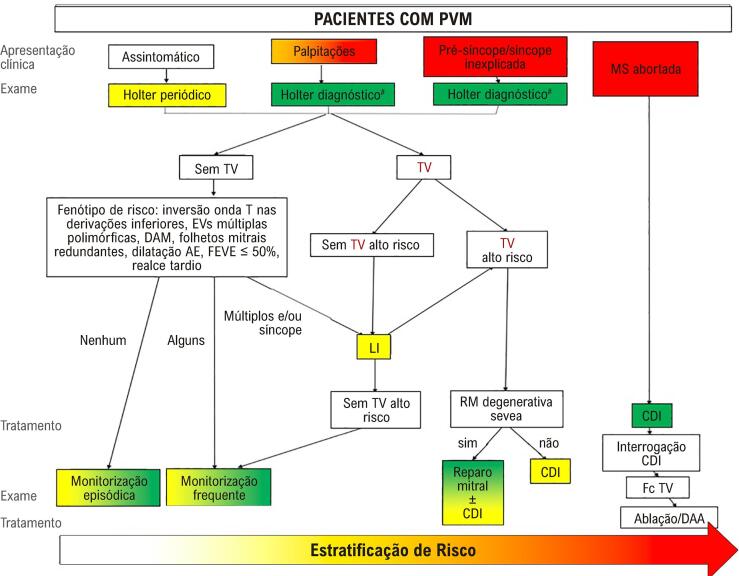
– Esquema de estratificação de risco nos pacientes com PVM. EVs: ectopias ventriculares; PVM: prolapso da válvula mitral; MS: morte súbita; CDI: cardiodesfibrilador implantável; AE: átrio esquerdo; FEVE: fração de ejeção de ventrículo esquerdo; DAM: disjunção do anel mitral; RM: regurgitação mitral; EVs: ectopias ventriculares; TV: taquicardia ventricular; LI: Looper implantável; DAA: droga antiarrítmica. #Método adicional de monitorização pode ser usado como Looper implantável. (Modificado de EHRA expert consensus statement on arrhythmic mitral valve prolapse and mitral annular disjunction complex).3

**Figura 3:**
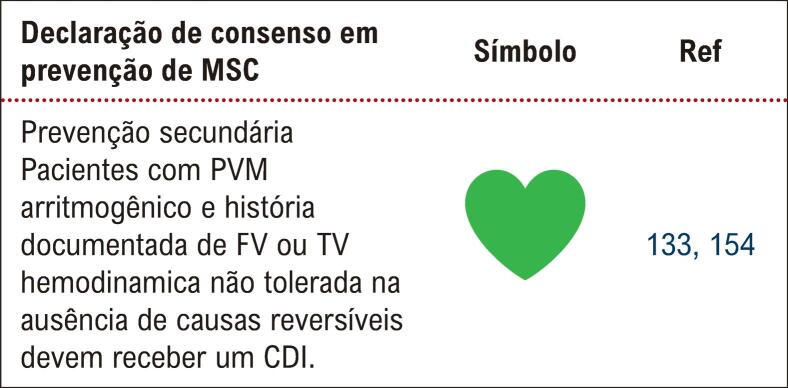
– Indicação de implante de CDI na prevenção secundária de morte súbita no PVM arritmogênico. (Modificado de EHRA expert consensus statement on arrhythmic mitral valve prolapse and mitral annular disjunction complex).3 PVM: prolapso da válvula mitral; FV: fibrilação ventricular; TV: taquicardia ventricular; CDI: cardiodesfibrilador implantável.

Com a utilização da definição atual, o PVM tem prevalência de 0,6 a 3,1%, dependendo da idade da população examinada (aumenta com a idade), e leve predominância no sexo feminino.^1^ Os desfechos clínicos são primariamente determinados pela severidade da regurgitação e suas consequências no tamanho e função do ventrículo esquerdo. A estratificação de risco pode ser feita baseada no contexto clínico associada ao ECG e ao tipo de arritmia encontrado. Na ausência de TV, os achados de fenótipo de risco irão ditar a intensidade de *screening* para a arritmia. As arritmias consideradas de risco são a TV sustentada, a TV não sustentada polimórfica, as arritmias rápidas (Fc >180 bpm) ou aquelas resultando em síncope. A Figura 2 ilustra o esquema de estratificação de risco nessa população. A MS e as arritmias ventriculares (AVs) podem ocorrer na ausência da regurgitação com prevalência de 4 a 7% na origem mixomatosa e 13% no subgrupo de mulheres. As arritmias complexas estão relacionadas a mortalidade independentemente do grau de regurgitação.^3^ Foi observado prolongamento do intervalo QTc ao Holter ECG 24h que era normal no ECG, método considerado mais adequado para essa medida. Embora a presença do aumento do intervalo QTc possa estar relacionada à Síndrome do QT longo, uma doença arrítmica primária que ocorre por alteração nos canais iônicos que afetam a repolarização, levando a *Torsades de Points* mediado por estresse físico ou emocional resultando em síncope ou MS, o prolongamento do intervalo QT no PVM, em geral, se correlaciona com prolapsos mais graves e está independentemente associado a AVs nessa população, muito embora a correlação com mortalidade nessa população seja incerta.^3 , 4^ A fragmentação do complexo QRS é um outro achado eletrocardiográfico que está associado a arritmias complexas no PVM. A prevalência da DAM no PVM varia de 20 a 58% e, entre aqueles com origem mixomatosa, varia de 21,8% a 98%. A DAM é um marcador de doença grave, incluindo mais eventos arrítmicos e maior necessidade de intervenção da válvula mitral.^3^ A paciente descrita foi diagnosticada com prolapso de válvula mitral arritmogênico e apresentou o pior cenário possível, que foi a MS recuperada, tendo indicação classe I de implante de CDI de acordo com a mais recente diretriz sobre o assunto ( figura 3 ).

## Conclusão

O não reconhecimento da entidade PVMA na internação após MS recuperada levou a um atraso na indicação do CDI, possibilitou a paciente ter uma gestação, e sofrer nova MS dois meses após o parto com desfecho desfavorável.
