# Supplementary material for: Morte Súbita em Lactante Portadora de Prolapso da Válvula Mitral Arritmogênico
Source: Arq Bras Cardiol. 2024 Mar 14;121(2):e20230358. [Article in Portuguese] doi: 10.36660/abc.20230358 (PMC12092033; doi:10.36660/abc.20230358)
Supplement: Translation

# Translation: Sudden Death in a Breastfeeding Woman with Arrhythmogenic Mitral Valve Prolapse

**Authors:** Erika Olivier Vilela Bragança, Fabio Luis Valério da Silva

**Affiliations:** 1 Brazil RitmoCheck, São José dos Campos, SP – Brazil

**Keywords:** Mitral Valve Prolapse, Sudden Death, Cardiac Arrhythmias

## Introduction

Mitral valve prolapse (MVP) is the most common valvular heart disease, affecting 2-3% of the general population, and is defined by the systolic displacement of one or both mitral valve leaflets ≥2 mm above the plane of the mitral annulus in the sagittal view of the mitral valve via echocardiography.^1^The outcomes of MVP without regurgitation are generally benign; however, a small, ill-defined subgroup of individuals is at high risk for malignant arrhythmias and sudden death (SD). Autopsy data show a 4% to 7% prevalence of MVP in young people with SD.^2^Due to the low event rate and lack of cohorts with a robust number of patients, accessing the accurate incidence of SD and MVP in the general population and in patient subgroups remains a challenge. The diagnosis of arrhythmogenic MVP is made in the presence of MVP and ventricular arrhythmia, considering a ventricular arrhythmia density ≥5% on 24-hour electrocardiogram (ECG) Holter or the presence of complex arrhythmia – non-sustained ventricular tachycardia (NSVT), ventricular tachycardia (VT) or ventricular fibrillation (VF), as well as in the absence of any other arrhythmogenic substrate.^3^

### Description

wWe report the case of JAS, a female, 27-year-old, married patient. Who was hospitalized 13 months ago after cardiorespiratory arrest (CPA), with a hospital discharge report that states “CPA in a shockable rhythm and successfully resuscitated”. The report also states hospitalization for 18 days, ECGs with no significant changes, echocardiogram with mild to moderate dilation of the left atrium, myxomatous mitral valve with significant prolapse of its leaflets associated with moderate valve insufficiency, moderate left ventricular dilation with left ventricular function preserved (left atrium diameter of 45 mm, left ventricular diastolic diameter of 66 mm and left ventricular ejection fraction of 67%) ( Figure 1A ) and coronary angiography with no obstructive lesions. The report concludes that “there is no need to prolong hospitalization for additional investigation and CPA of undetermined cause after investigation”. The patient was discharged from the hospital without medication and was advised to seek medical attention for follow-up. As complications during hospitalization, the patient presented bronchoaspiration pneumonia, renal failure that required hemodialysis, and ischemic stroke in the left parietal region.

**Figure 1:** – A) Echocardiogram: color Doppler of the mitral valve with significant regurgitation. Ventricular dilation is observed; B) 12-lead electrocardiogram: sinus rhythm, 1 degree interatrial block (P wave duration 125 ms), R wave fragmentation in leads D1 and aVL, QTc interval of 420 ms, ventricular ectopia with right bundle branch block morphology, with superior axis (D2 and D3 negative), ectopic QRS complex predominantly negative in lead V5, and ectopic QRS duration of 195 ms - suggestive of origin in the posterior papillary muscle; C) Magnetic resonance imaging of the heart: significant left dilatation, mitral valve prolapse with severe regurgitation and disjunction of the mitral annulus.

After hospital discharge, the patient presented irregular menstrual cycles, was not taking birth control, and became pregnant. She was primiparous, G1P0A0, and the pregnancy was uneventful. She underwent a Cesarean section at 38 weeks, as recommended by her cardiologist, and gave birth to a female baby, weighing 2,600 grams, with a height of 47 centimeters, and Apgar scores of 8 and 10. She was discharged from the hospital within 48 hours. She came to the first and only consultation with her 2-week-old breastfed baby, accompanied by her 13-year-old niece, complaining of tiredness with moderate exertion. As a personal medical history, she reported arrhythmia since childhood and the other medical conditions mentioned above in the hospital discharge report. She had no relevant family history, was not a smoker and/or alcohol drinker, did not exercise, refused other surgeries, did not have COVID-19, and did not regularly use medication. During the physical examination, it was noted that the patient was breastfeeding and on cardiac auscultation, a systolic murmur was detected in the mitral focus +/4+ radiating to the axilla, but the rest was normal. The ECG showed a sinus rhythm, 1 degree interatrial block (P wave duration 125 ms), R wave fragmentation in leads D1 and aVL, QTc interval of 420 ms, ventricular ectopy with right bundle branch block morphology, with superior axis (D2 and D3 negative), ectopic QRS complex predominantly negative in lead V5, and ectopic QRS duration of 195 ms, which is suggestive of origin in the posterior papillary muscle ( Figure 1B ). She presented her transthoracic echocardiogram, described above, and the image was suggestive of significant mitral regurgitation with dilation of the left ventricle ( Figure 1A ). She also presented a 24-hour ECG Holter with frequencies that varied from a minimum of 42 bpm, an average of 68 bpm, and a maximum of 113 bpm, 2,449 (3%) bimorphic ventricular ectopias, of which 2,428 were isolated, with 9 pairs and 1 NSVT of 3 beats and frequency of 121 bpm; 1 atrial ectopia was isolated, with diffuse changes in ventricular repolarization with prolongation of the QTc interval (maximum QTc 500 ms) and no correlation between symptoms and electrocardiographic changes. The diagnostic hypothesis was arrhythmogenic MVP (AMVP) in a patient recovered from SD and the implantable cardioverter defibrillator (ICD) and mitral valve repair were indicated. The patient refused treatment, despite all the instructions given, and requested to return with her husband for a scheduled appointment. Beta-blocker treatment was begun and magnetic resonance imaging (MRI) of the heart was requested. On the appointment date, one month later, the patient rescheduled, requesting another month to return. One week before the scheduled date, the patient developed a new cardiac arrest and resuscitation was attempted without success. Before the consultation, the patient had undergone heart MRI ( Figure 1C ). The report was obtained post-mortem at the clinic that performed the examination and showed enlargement of the left atrium, dilation of the left ventricle associated with MVP and disjunction of the mitral annulus (MAD), severe and eccentric mitral regurgitation, and increased subendocardial trabeculation associated with anterolateral and inferolateral basal and medial subendocardial fibrosis.

## Discussion

Using the current definition, MVP has a prevalence of 0.6 to 3.1%, depending on the age of the population examined (increases with age), and a slight predominance in females.^1^Clinical outcomes are primarily determined by the severity of regurgitation and its consequences on the size and function of the left ventricle. Risk stratification can be done based on the clinical context associated with the ECG and the type of arrhythmia found. In the absence of VT, the risk phenotype findings will dictate the intensity of screening for the arrhythmia. The arrhythmias considered at risk are sustained VT, polymorphic non-sustained VT, rapid arrhythmias (HR >180 bpm), or those resulting in syncope. Figure 2 illustrates the risk stratification scheme in this population. SD and ventricular arrhythmias (VAs) can occur in the absence of regurgitation with a prevalence of 4% to 7% in myxomatous origin and 13% in the subgroup of women. Complex arrhythmias are related to mortality regardless of the degree of regurgitation.^3^Prolongation of the QTc interval was observed on the 24-hour ECG Holter, which was normal in the ECG, a method considered more appropriate for this measurement. Although the presence of an increase in the QTc interval may be related to Long QT Syndrome, a primary arrhythmic disease that occurs due to changes in ion channels that affect repolarization, leading to Torsades de Points mediated by physical or emotional stress, resulting in syncope or SD, QT interval prolongation in MVP generally correlates with more severe prolapses and is independently associated with VAs in this population, although the correlation with mortality in this population is uncertain.^3 , 4^Fragmentation of the QRS complex is another electrocardiographic finding that is associated with complex arrhythmias in the MVP. The prevalence of mitral annular disjunction in MVP varies from 20% to 58% and, among those with myxomatous origin, it varies from 21.8% to 98%. MAD is a marker of serious disease, including more arrhythmic events and a greater need for mitral valve intervention.^3^The patient described was diagnosed with AMVP and presented the worst possible scenario, which was recovered SD, indicating a class I ICD implantation according to the most recent guideline on the subject ( Figure 3 ).

**Figure 2:** – Risk stratification scheme in patients with MVP. PVCs: premature ventricular contractions; MVP: mitral valve prolapse; SD: sudden death; ICD: implantable cardioverter defibrillator; LA: left atrium; LVEF: left ventricular ejection fraction; MAD: mitral annular disjunction; MR: mitral regurgitation; VEs: ventricular ectopias; VT: ventricular tachycardia; IL: Implantable looper; AAD: antiarrhythmic drug. #Additional monitoring method can be used as deployable Looper. (Modified from EHRA expert consensus statement on arrhythmic mitral valve prolapse and mitral annular disjunction complex).3

**Figure 3:** – Indication for ICD implantation in the secondary prevention of sudden death in arrhythmogenic MVP. (Modified from EHRA expert consensus statement on arrhythmic mitral valve prolapse and mitral annular disjunction complex).3 MVP: mitral valve prolapse; VF: ventricular fibrillation; VT: ventricular tachycardia; ICD: implantable cardioverter defibrillator.

## Conclusion

The non-recognition of the AMVP entity upon admission after recovered SD led to a delay in the indication of the ICD, allowing the patient to have a pregnancy, and subsequently suffer a new SD two months after delivery with an unfavorable outcome.
